# Hematopoietic Stem Cell Transplantation in Inborn Errors of Metabolism

**DOI:** 10.3389/fped.2019.00433

**Published:** 2019-10-25

**Authors:** Emily Y. Tan, Jaap Jan Boelens, Simon A. Jones, Robert F. Wynn

**Affiliations:** ^1^Faculty of Health and Medical Sciences, University of Western Australia, Perth, WA, Australia; ^2^Stem Cell Transplant and Cellular Therapies, Memorial Sloan Kettering Cancer Center, New York, NY, United States; ^3^Metabolic and Blood and Marrow Transplant Units, Royal Manchester Children's Hospital, Manchester, United Kingdom

**Keywords:** hematopoietic stem cell transplantation, bone marrow transplant, inborn errors of metabolism, lysosomal storage disease, peroxisomal disease, mitochondrial disease

## Abstract

Hematopoietic stem cell transplantation (HSCT) has been established as an effective therapy for selected inborn errors of metabolism. The success of HSCT in metabolic disease is best exemplified through the treatment of Hurler's syndrome, a lysosomal storage disease. Through the collaborative effort of several international centers, factors that predict successful patient and transplant outcomes have been identified. In this review, we discuss the principles that underlie the use of HSCT in metabolic diseases. We consider the clinical indications, conditioning regimens, and disease-specific follow-up for HSCT in different metabolic diseases. We highlight persisting challenges in HSCT to delay progression of certain organ systems that remain refractory to HSCT and the relatively high rates of aplastic graft failure. Finally, we evaluate the variable applicability of these principles to other inherited metabolic disorders including peroxisomal, mitochondrial, and other lysosomal storage diseases.

## Key Messages

1. Hematopoietic Stem Cell Transplantation (HSCT) for metabolic diseases is specialized medicine. It requires multidisciplinary management and continuous collaboration with other specialties and allied health professionals before, during and beyond the period of transplantation.
2. HSCT is better at preventing disease progression than in reversing already established disease manifestations.
3. Early intervention with HSCT is associated with better outcomes.
4. Transplant outcomes should be distinguished from disease outcomes in metabolic disease, and much progress has been made in recent years from careful, multi-center and collaborative analysis of the factors that separately influence each.
5. Engraftment rates are improved with pharmacokinetic analysis-guided, myeloablative busulfan without *ex-vivo* T-cell depletion.
6. Autologous HSCT using gene therapy may provide an improved treatment option for inherited metabolic diseases in the future, both by reducing allogeneic treatment-related toxicities, and by improving efficacy through augmented graft enzyme delivery.

## Introduction

Inherited metabolic disorders comprise a large, diverse, and complex group of diseases caused by defects in genes that code for proteins involved in metabolic pathways. HSCT is an option and even standard of care for specific metabolic diseases, where other available therapies are less effective and where the benefit of HSCT outweighs the risk of a transplant. This chapter will serially discuss the use of HSCT in certain lysosomal storage and peroxisomal diseases where HSCT is standard of care. Furthermore, it will discuss its conditional role in other metabolic disease including mitochondrial disease (Table 1).

**Table 1:** Inherited metabolic disorders where HSCT may be indicated.

**Disorder**	**Enzyme/protein**	**Indication**	**Comments**
**LYSOSOMAL STORAGE DISEASES**			
**Mucopolysaccharidoses**			
Hurler (MPS-IH)	Alpha-L-iduronidase	Standard	
Attenuated MPSI	Alpha-L-iduronidase	Option	ERT first-line therapy
Hunter: severe (MPS-IIA)	Iduronate-2-sulfatase	Investigational	Only early or asymptomatic, and ERT is often used for somatic disease in these patients
Hunter: attenuated (MPS-IIB)	Iduronate-2-sulfatase	Option	ERT first-line therapy
Maroteaux-Lamy (MPS-VI)	Arylsulfatase B	Option	ERT first-line therapy
Sly (MPS-VII)	Beta-glucuronidase	Option	ERT just licensed
**Sphingolipidoses**			
MLD: late infantile	Arylsulfatase A	Standard	Gene therapy is standard
MLD: early juvenile	Arylsulfatase A	Option	Consider gene therapy as option
MLD: late juvenile	Arylsulfatase A	Option	Consider gene therapy as option
MLD: adult onset	Arylsulfatase A	Standard	Only early or asymptomatic
GLD: early onset	Galactocerebrosidase	Option—only if patient is diagnosed in first weeks of life, is asymptomatic and family understands there will be significant disease manifestations	
GLD: late onset	Galactocerebrosidase	Standard	Only early or asymptomatic
Niemann pick: Type A	Acid sphingomyelinase	Investigational	
Niemann pick: Type B	Acid sphingomyelinase	Investigational	ERT available
Niemann pick: Type C1, C2	Cholesterol trafficking	No	Does not correct neurological progression even in C2
GM2 Gangiosidosis (Tay Sachs and Sandhoff): early onset	Hexosaminidase A and B	No	
GM2 Gangiosidosis (Tay Sachs and Sandhoff): late onset	Hexosaminidase A and B	Option	In known family
Farber	Ceramidase	Option	Especially for somatic disease
**Glycoproteinoses**			
Alpha-mannosidosis	Alpha-mannosidase	Option	
Fucosidosis	Fucosidase	Option	
Aspartylglucosaminuria	Aspartylglucosaminidase	Option	
**Other**			
Multiple sulfatase deficiency	Sulfatases	Investigational	Really no evidence to support transplant
Wolman syndrome	Lysosomal acid lipase	Option	ERT is likely first line
Pompe	Glucosidase	Investigational	ERT first-line therapy
**PEROXISOMAL DISEASES**			
X-ALD, cerebral	ALD protein	Standard in early phase of childhood cerebral inflammatory disease	No advanced disease, gene therapy option in trial
**MITOCHONDRIAL DISEASES**			
MNGIE	Thymidine phosphorylase	Option	No advanced disease, including minimal gastrointestinal involvement

## Lysosomal Storage Diseases

### Pathophysiology

The Lysosomal Storage Diseases (LSDs) encompass over 70 diseases, which comprise genetic defects in specific lysosomal proteins. In the past decade, the function of lysosomes has extended beyond their involvement in degradation and recycling of extracellular and intracellular material. They play a crucial role in plasma membrane repair, lipid and metabolite exchange between organelles and have recently been found to regulate energy metabolism via calcium signaling (1–3). Understandably, genetic defects in lysosomal proteins involved in any of these microdomains can have broad functional consequences. The pathogenesis of cellular injury is not fully understood but stems from the primary accumulation of undigested substrates within lysosomes and subsequent downstream pathology (4).

Mucopolysaccharidosis Type 1 (MPS I) is an autosomal recessive LSD characterized by lysosomal accumulation of mucopolysaccharides or glycosaminoglycans (GAGs) (5). In MPS I, patients have a defective mutation in the *IDUA* gene which codes for alpha-l-iduronidase, resulting in ineffective catabolism of heparan and dermatan sulfate (5). Accumulation and subsequent deposition of these GAGs in vital organs causes significant multiorgan dysfunction. This can manifest as progressive mental retardation, skeletal deformities, gastrointestinal pathology, and visual and auditory impairment (6). The clinical severity of MPS I is observed across a vast spectrum. MPS IH, or Hurler's Syndrome, is the more severe phenotype of MPS I where patients have an early-onset, rapidly progressive disease with neurological involvement. In untreated children with MPS IH, death is usual in the first decade of life, often from cardiac or respiratory complications (7, 8).

### Indication for HSCT

LSDs require early intervention and multi-disciplinary management to optimize treatment response, quality of life and prevent premature mortality. The principle of HSCT in LSDs is in cross-correction. HSCT provides the recipient with a continuous source of enzyme produced by donor-derived myeloid cells, which are then taken up by enzyme-deficient host cells (9). Furthermore, the superiority of HSCT to enzyme replacement therapy (ERT) lies in its exploitation of donor-derived cells to migrate across the blood brain barrier and differentiate into tissue macrophages, known as microglia, which secrete the deficient enzyme to the central nervous system, improving neurocognitive outcomes (10). MPS IH is the paradigm of successful HSCT in metabolic disease. HSCT is the gold-standard treatment option for MPS IH patients who are younger than 2 years of age who have no or minimal cognitive impairment (11). Currently available ERT is ineffective in preventing cognitive decline as it is unable to cross the blood brain barrier in sufficient doses and long-term therapy with ERT is limited by the induction of anti-enzyme antibodies, diminishing substrate reduction (10, 12–14).

### Approach to HSCT, Outcomes and Disease-Specific Follow-Up

#### Conditioning

Full intensity myeloablative conditioning with fludarabine and pharmacokinetic-guided busulfan dosing is the current recommendation for LSDs (15). Parenteral busulfan with therapeutic drug monitoring has facilitated more precise dose delivery (16, 17). This has mitigated previously high incidences of hepatic veno-occlusive disease (VOD) associated with increased busulfan exposure, while ensuring adequate therapeutic levels are achieved to avoid graft rejection (18). Furthermore, although cyclophosphamide (CY) was originally used instead of fludarabine, the readily described CY-associated cardiac toxicity and reduced duration of neutropenia with fludarabine, as well as reduced rates of VOD, has limited the use of CY in pre-transplant conditioning (19) (see the review “Conditioning Perspectives for Primary Immunodeficiencies”).

#### Transplant Outcomes

In the past two decades, the proportion of MPS IH patients with graft failure has declined by more than 3-fold (20). Preferential use of umbilical cord blood (UCB) has shown superiority in achieving full-donor chimerism, where an increased number of patients have more than 95% of donor-derived haematopoiesis, compared to other cell sources (20, 21). The interval to transplant is reduced in UCB transplant, and there is better tolerance of HLA-mismatch. In addition, UCB is associated with greater delivery of normal enzyme levels, improving disease-related outcomes (17, 22). Some optimization of the UCB transplant procedure is required since there remain relatively high rates of aplastic graft failure requiring re-transplant, and of immune-mediated cytopenia (IMC) (20, 23). Assessment and optimization of contributing factors to graft failure and IMC is an active area of investigation.

#### Patient Outcomes

HSCT undoubtedly improves the clinical course of patients with MPS IH. Following HSCT, early disease-related mortality is reduced and prolonged survival possible and even expected (24, 25). However, there remain disease manifestations that require long term follow up. Earlier age at HSCT and graft enzyme output are the twin predictors of superior clinical outcomes in MPS IH, including preservation of neurocognition (24). Somatic outcomes including cardiac disease and corneal clouding stabilizes and has shown improvement in the majority of MPS IH patients post-transplant (24). In contrast, orthopedic complications continue to progress despite transplant, with the majority of patients requiring surgical intervention (24). Thus, the capacity for HSCT to ameliorate disease progression varies widely between organ systems, and certain organs remain relatively refractory to HSCT. Enzyme delivery after transplant may be augmented using an *ex vivo* stem cell gene therapy procedure and this may improve skeletal outcomes.

#### Follow Up

Multidisciplinary management beyond the period of transplantation is fundamental in the care of patients with LSD. HSCT improves the clinical course of disease but it is not curative. Regular, long-term follow up with several specialties is mandatory including a bone marrow transplant clinician, metabolic disease specialist, endocrinologist, orthopedic specialist, and spinal surgeon. The psychosocial impact of the disease should not be neglected and psychological support should be offered.

### The Role of HSCT in Other LSDs

MPS IH has exemplified core underlying principles of HSCT in LSDs. It has demonstrated the concept of efficacious enzyme delivery via cross-correction, facilitated optimization of a cell source hierarchy that includes UCB, and optimization of a fludarabine and pharmacokinetic-guided busulfan conditioning regimen. It has highlighted the importance of performing HSCT early and pre-symptomatically. While some of these principles are applicable to a few LSDs, it is not indicated in others as it fails to improve patient outcomes.

In Alpha Mannosidosis (AM), HSCT is considered standard of care based on limited published evidence (26). Deficiency of alpha-mannosidase causes a clinically heterogenous disease of neurocognitive impairment and musculoskeletal abnormalities (27, 28). In AM, earlier age at HSCT predicts superior clinical outcomes (29). Similarly, it was thought that early, pre-symptomatic HSCT was crucial for altering the course of rapid neurological disease in infantile Globoid Cell Leukodystrophy (GLD) (30). However, the introduction of newborn screening (NBS) in New York yielded controversial patient outcomes, showing significant HSCT-associated morbidity and mortality (31). Long term collaborative multi-center data may provide better insight into the utility of HSCT in GLD.

There is no role for HSCT in Sanfilippo syndrome (MPS III) or infantile Metachromatic Leukodystrophy (MLD). MLD is caused by deficiency of arylsulfatase A and subsequent accumulation of sulfatides in the central and peripheral nervous system, resulting in widespread demyelination (32, 33). What has been learnt from MPS IH has not reliably translated to infantile MLD or MPS III. Despite pre-symptomatic HSCT and utilization of UCB, HSCT does not halt disease progression in infantile MLD (brain seems modified, but peripheral nerve system not) or MPS III (34–36). Patient outcomes for infantile MLD following HSCT is complicated by peripheral neuropathy and significant HSCT-associated morbidity (36, 37). Even with full engraftment in MPS III patients, there is no biochemical correction of the disease in the CSF (35). In MLD, transplant failure may be largely attributable to the slow and gradual replacement of resident tissue macrophages and microglia populations by donor-derived progeny compared with the rapid progression of disease. Furthermore, donor-derived microglial cells may secrete insufficient amounts of enzyme to correct neuronal tissue in these LSDs. *Ex-vivo* stem cell gene therapy of autologous HSC improves graft enzyme delivery and has been shown to be dramatically beneficial in modifying disease progression in infantile MLD (38) (see the review “Autologous stem cell-based gene therapy for inherited disorders: state-of-the-art and future prospects”).

## Peroxisomal Diseases: X-ALD

### Pathophysiology

Adrenoleukodystrophy (X-ALD) is caused by a genetic mutation in *ABCD1*, resulting in a deficiency of ALD, a membrane transporter which transports substrates from the cytosol into the peroxisome (39). Subsequently, very long-chain fatty acids (VLCFA) are insufficiently degraded via peroxisomal beta-oxidation, accumulate in the cytosol, and incorporate into different complex lipids in varying degrees across cell types, although the nervous system, and adrenal glands are particularly vulnerable (40, 41). The vast phenotypic variation of X-ALD can be simplified into four categories; asymptomatic, adrenal failure, adrenomyeloneuropathy, and inflammatory cerebral disease (42). Childhood cerebral ALD (CCALD) is the most severe manifestation, associated with rapid neurological decline and mortality if untreated (42). The initial episode of cerebral demyelination that occurs with CCALD could be attributed to selective accumulation of VLCFA in myelin, leading to progressive destabilization of myelin sheaths and subsequent demyelination (43). Complex lipids containing VLCFA may cause microglial activation, apoptosis, and diminish their capacity to oxidize VLCFA or provide neuroprotective factors for neighboring cells, leading to rapidly progressive inflammatory demyelination (44, 45).

### Indication for HSCT

HSCT is only indicated in CCALD and is the single treatment modality available in this phenotype to demonstrate amelioration of disease (46). The efficacy of HSCT in X-ALD is unlikely to be in cross-correction. HSCT may arrest the neuroinflammatory demyelinating process by replacing dysfunctional microglia with bone-marrow derived macrophages but this mechanism has not been fully elucidated (47).

### Approach to HSCT, Outcomes and Disease-Specific Follow-Up

Myeloablative conditioning with busulfan and CY with or without total body irradiation (TBI) has originally been used for HSCT in X-ALD (48). Recently, a reduced intensity conditioning regimen with fludarabine, melphalan, and low-dose TBI has been trialed, demonstrating promising survival rates and modest neurological stabilization, both clinically and on imaging (49, 50). Optimal patient outcomes are achieved in patients with limited clinical evidence of cerebral disease at HSCT (48, 51). To reduce time to transplant, NBS may facilitate early detection of HSCT-eligible individuals and identification of transplant donors which can be recruited once the need for HSCT manifests (52).

Regular evaluation with multi-disciplinary input is necessary to provide appropriate assessment and management for the diverse complications that may arise with X-ALD. This includes follow-up by an endocrinologist, neurologist, and ongoing radiological monitoring by MRI to detect early cerebral disease in genetically affected boys, or evaluate neurological response to treatment.

## Mitochondrial Diseases: MNGIE

Mitochondrial neurogastrointestinal encephalomyopathy (MNGIE) is an autosomal recessive disease characterized by deficiency of thymidine phosphorylase (TP) (53). Nucleosides accumulate and cause mitochondrial DNA instability (54). This manifests as a constellation of clinical features that includes peripheral neuropathy, external ophthalmoplegia, gastrointestinal dysmotility, and leukoencephalopathy (53). HSCT may pose as a viable treatment option where donor-derived normalization of TP enzymatic activity can eliminate accumulated nucleosides. While there is limited published evidence for HSCT, the results are promising and suggest that HSCT should only be considered in younger patients before severe gastrointestinal dysmotility develop and only if a fully matched donor is available (55, 56).

## Summary and Future Perspectives

- The collaborative effort of several international studies has led to the success of HSCT in treating metabolic disease, particularly MPS IH.
- The principle of cross-correction does not reliably translate to all LSDs or other metabolic diseases, and persistent progression of organs that are refractory to HSCT cause significant morbidity following transplant.
- Autologous HSCT using gene therapy may facilitate supranormal enzyme production by transducing a patient's HSCs with a viral vector *ex vivo*, and infusing them back into the patient following appropriate conditioning.
- These gene-corrected cells can integrate into the host genome, where certain viral vectors such as lentiviruses can do so with a lower risk of insertional mutagenesis.
- This may help treat refractory diseases such as MLD and MPSIIIA, and better correct refractory organs in a responding disease, such as the skeleton in MPSIH, while avoiding significant HSCT-associated morbidity. Pre-clinical experiments in animal models are promising and clinical trials are ongoing.

## Author Contributions

ET and RW were the main authors with contributions by JB and SJ. All authors reviewed the manuscript.

### Conflict of Interest

The authors declare that the research was conducted in the absence of any commercial or financial relationships that could be construed as a potential conflict of interest.
